# Supplementary material for: Anti-neuraminidase and anti-HA stalk antibodies reduce the susceptibility to and infectivity of influenza A/H3N2 virus
Source: Nat Commun. 2025 Dec 11;16:10910. doi: 10.1038/s41467-025-65283-0 (PMC12698684; doi:10.1038/s41467-025-65283-0)
Supplement: Supplementary file 1 — Supplementary Information [file 41467_2025_65283_MOESM1_ESM.pdf]

Supplement For: Anti-Neuraminidase and anti-HA stalk Antibodies Reduce the Susceptibility to and Infectivity of Influenza A/H3N2 Virus

Gregory Hoy[1+], Thomas Cortier[2,3+], Hannah E. Maier[1], Guillermina Kuan[4,5], Roger Lopez[4,6], Nery Sanchez[4], Sergio Ojeda[4], Miguel Plazaola[4], Daniel Stadlbauer[7], Abigail Shotwell[1], Angel Balmaseda[4,6], Florian Krammer[7,8,9,10], Simon Cauchemez[1*], and Aubree Gordon[1*]

[1]School of Public Health, University of Michigan, Ann Arbor, Michigan, USA; [2]Mathematical Modelling of Infectious Diseases Unit, Institut Pasteur, Université Paris Cité, INSERM U1332, CNRS UMR2000, Paris, France; [3]Collège Doctoral, Sorbonne Université, Paris, France; [4]Sustainable Sciences Institute, Managua, Nicaragua; [5]Centro de Salud Sócrates Flores Vivas, Ministry of Health, Managua, Nicaragua; [6]Laboratorio Nacional de Virología, Centro Nacional de Diagnóstico y Referencia, Ministry of Health, Managua, Nicaragua [7]Department of Microbiology, Icahn School of Medicine at Mount Sinai, New York, New York, USA [8]Center for Vaccine Research and Pandemic Preparedness (C-VaRPP), Icahn School of Medicine at Mount Sinai, New York, NY, USA. [9]Department of Pathology, Molecular and Cell-Based Medicine, Icahn School of Medicine at Mount Sinai, New York, NY, USA. [10]Ignaz Semmelweis Institute, Interuniversity Institute for Infection Research, Medical University of Vienna, Vienna, Austria

+: Equal contribution

*: Denotes co-senior authors

Table of Contents

1 Supplementary tables 3

1.1 Details about the antigens used for immunologic assays 3

1.2 Sensitivity Analysis on Prior Assumptions 3

1.3 Sensitivity analysis on disease life history traits 4

1.4 Simulation study of the model to assess parameter identifiability 6

1.5 Gelman-Rubin Diagnostic Results 8

1.6 Descriptive statistics on infected cases according to their potential place of infection 9

2 Supplementary figures 10

2.1 Complete model including the estimate of infectivity for HAI 10

2.2 Estimates of the contact rate per household size and the relative ability of transmission of influenza during the first wave compared to the second and the third wave 12

2.3 Necessity of Robust Antibody Responses to Reduce Susceptibility and Infectivity 13

2.4 Assessing model adequacy comparing observed SAR and SAR within model simulations 16

2.5 Assessing bias in parameter estimates comparing the posterior distributions from chains on simulated datasets to the original parameter values 17

2.6 Correlation between antibody titers through scatter plot representation 18

3 Supplementary Methods 20

3.1 Data considered in statistical models 20

3.2 XGBoost Framework 20

3.2.1 Details about the statistical tool 20

3.2.2 The definition of the model 21

3.3 The transmission model 23

3.3.1 The basal transmission model 23

The association between the titer and the individual susceptibility 24

The association between the titer and the individual infectivity 25

3.3.2 Risk of infection of contact j 26

3.3.3 The probability of transmission from the community 26

3.3.4 The likelihood of the transmission process in household 26

3.3.4.1 The contribution to the likelihood of a uninfected individuals 27

3.3.4.2 The likelihood of an infected household contact 27

3.3.4.3 The likelihood of symptom onset and test time given infection time 27

3.3.5 MCMC inference algorithm 28

3.3.6 Sensitivity on virus life history hypothesis and prior distributions 28

3.3.6.1 Disease Life History Traits 29

3.3.6.2 Sensitivity Analysis on Prior Assumptions 29

3.3.7 Programming 30

1 Supplementary tables

1.1 Details about the antigens used for immunologic assays

Assay	Antigen
Hemagglutination inhibition assay	A/Hong Kong/4801/2014
H3 ELISA	A/Hong Kong/4801/2014
N2 ELISA	A/Hong Kong/4801/2014
Stalk ELISA	cH7/3 with A/Anhui/1/2013 as the head domain and A/Hong Kong/4801/2014 as the stalk domain

Supplementary Table 1 Antigens used for immunologic assays

1.2 Sensitivity Analysis on Prior Assumptions

We tested the robustness of our results to different prior distributions for susceptibility ratios and antibody thresholds. Results remained consistent across scenarios.

Parameter	NA susceptibility	HAI susceptibility	Stalk susceptibility	NA infectivity	HAI infectivity
Default model	0.49 (0.30-0.75)	0.63 (0.42-0.98)	0.66 (0.44-0.99)	0.55 (0.32-0.98)	0.53 (0.27-0.97)

Factor prior SD 2.0	0.48 (0.28-0.76)	0.64 (0.43-0.95)	0.67 (0.45-1.00)	0.52 (0.31-0.90)	0.50 (0.23-0.95)
Threshold prior width = 20	0.50 (0.21-0.78)	0.59 (0.41-0.86)	0.65 (0.44-0.95)	0.51 (0.31-0.86)	0.55 (0.28-0.97)
Threshold prior width = 40	0.50 (0.32-0.77)	0.62 (0.42-0.95)	0.66 (0.45-0.95)	0.54 (0.32-0.99)	0.53 (0.25-0.98)
Threshold prior width = 60	0.48 (0.30-0.78)	0.64 (0.4-0.97)	0.65 (0.44-0.99)	0.54 (0.32-1.04)	0.54 (0.26-1.11)
Threshold prior width = 80	0.49 (0.30-0.76)	0.62 (0.43-0.96)	0.66 (0.43-1.03)	0.53 (0.32-1.04)	0.55 (0.26-1.17)

Supplementary Table 2 Sensitivity analyses testing for prior assumptions

1.3 Sensitivity analysis on disease life history traits

**Parameter**	**NA susceptibility**	**HAI susceptibility**	**Stalk susceptibility**	**NA infectivity**	**Stalk infectivity**
Default model	0.49 (0.30-0.75)	0.63 (0.42-0.98)	0.66 (0.44-0.99)	0.55 (0.32-0.98)	0.53 (0.27-0.97)

Incubation 0.6 days (SD 0.8)	0.50 (0.31-0.87)	0.64 (0.43-0.94)	0.64 (0.43-0.95)	0.51 (0.32-0.87)	0.54 (0.26-0.97)
Incubation 2 days (SD 1.6)	0.46 (0.27-0.74)	0.63 (0.42-0.98)	0.68 (0.45-1.04)	0.58 (0.35-1.01)	0.53 (0.26-0.97)
Infectivity 3.0 days (SD 1.5)	0.49 (0.30-0.75)	0.64 (0.43-0.96)	0.65 (0.43-0.99)	0.57 (0.34-0.99)	0.59 (0.29-1.07)
Infectivity 4 days (SD 2.0)	0.49 (0.30-0.76)	0.64 (0.43-0.95)	0.67 (0.4-0.99)	0.51 (0.31-0.90)	0.49 (0.25-0.91)

Supplementary Table 3 Sensitivity analyses testing for disease traits (generation time and incubation time)

1.4 Simulation study of the model to assess parameter identifiability

Parameter	Simulation value	Mean estimate	Proportion covered
α community infectious risk	0.000412	0.000666	94%
β baseline household force of infection	0.330	0.319	96%
δ household size dependency	0.549	0.583	94%
Rs_{NA} NA susceptibility	0.49	0.48	95%
Rs_{HAI} HAI susceptibility	0.64	0.64	96%
Rs_{Stalk} Stalk susceptibility	0.67	0.64	95%
Ks_{NA} NA susceptibility threshold	32	33	97%
Ks_{HAI} HAI susceptibility threshold	32	35	99%
Ks_{Stalk} Stalk susceptibility threshold	35	36	98%
Ri_{NA} NA infectivity	0.55	0.58	95%
Ri_{Stalk} Stalk infectivity	0.53	0.57	96%
Ki_{NA} infectivity threshold	27	28	99%

Ki_{Stalk} Stalk infectivity threshold	58	57	99%
ρ_{age} children susceptibility	2.54	2.64	96%
μ_{age} children infectivity	1/1.73	1/1.91	95%
κ first was relative transmission	0.71	0.72	96%

Supplementary Table 4: Simulation study of our baseline model. The first column is the simulation parameter name and type. The second column is the value of the parameter used in the dataset simulation. The third column is the mean of parameters estimated. The fourth column is the proportion of 95% credible intervals containing the simulation value.

1.5 Gelman-Rubin Diagnostic Results

We used Gelman–Rubin statistics $\hat{R}$ to assess MCMC chain convergence for each parameter. Estimates were computed from 20 independent chains with random starting points and different seeds, using the R package coda.

**Parameter**	Point est.	Upper C.I
α community infectious risk	1.001	1.001
β baseline household force of infection	1.000	1.000
δ household size dependency	1.000	1.001

Rs_{NA} NA susceptibility	1.001	1.003
Rs_{HAI} HAI susceptibility	1.001	1.002
Rs_{Stalk} Stalk susceptibility	1.001	1.002
Ks_{NA} NA susceptibility threshold	1.000	1.001
Ks_{HAI} HAI susceptibility threshold	1.001	1.003
Ks_{Stalk} Stalk susceptibility threshold	1.000	1.001
Ri_{NA} NA infectivity	1.001	1.002
Ri_{Stalk} Stalk infectivity	1.001	1.001
Ki_{NA} infectivity threshold	1.000	1.000
Ki_{Stalk} Stalk infectivity threshold	1.000	1.000
ρ_{age} children susceptibility	1.000	1.000
μ_{age} children infectivity	1.000	1.000
κ first was relative transmission	1.001	1.003

Supplementary Table 5: Gelman–Rubin estimates with upper confidence bounds. The first column indicates the simulation parameter name and type. The second column shows the estimated Gelman–Rubin statistic $\hat{R}$. The third column presents the 95% upper confidence interval of the estimate.

1.6 Descriptive statistics on participants

	**Infected**<br>Number (%)<br>Median (sd)	**Not infected**<br>Number (%)<br>Median (sd)	**All**<br>Number (%)<br>Median (sd)	**P-value**
Vaccination				
Ever vaccinated	40 (12.5)	36 (7.0)	76 (9.1)	0.009
Recently Vaccinated*	2 (0.6)	0 (0.0)	2 (0.2)	NA
Season				
2014-2015	44 (13.8)	90 (17.4)	134 (16.0)	0.1135
2016-2017	122 (38.2)	164 (31.8)	286 (34.3)	
2017-2018	153 (48.0)	262 (50.8)	415 (49.7)	
Stalk	26 (68)	55 (168)	40 (140)	1e-15
HA head	40 (246)	80 (387)	40 (341)	2e-5
NA	36 (284)	98 (970)	70 (785)	<2e-16

Supplementary Table 6: Descriptive statistics by infection status. The first column includes infected individuals, the second uninfected individuals, and the third all participants. For categorical variables, we report counts and percentages, with proportions compared using a test of proportion. For continuous variables, we report the

median and standard deviation, and comparisons are based on a two-sided Mann–Whitney U test.

2 Supplementary figures

2.1 Complete model including the estimate of infectivity for HAI

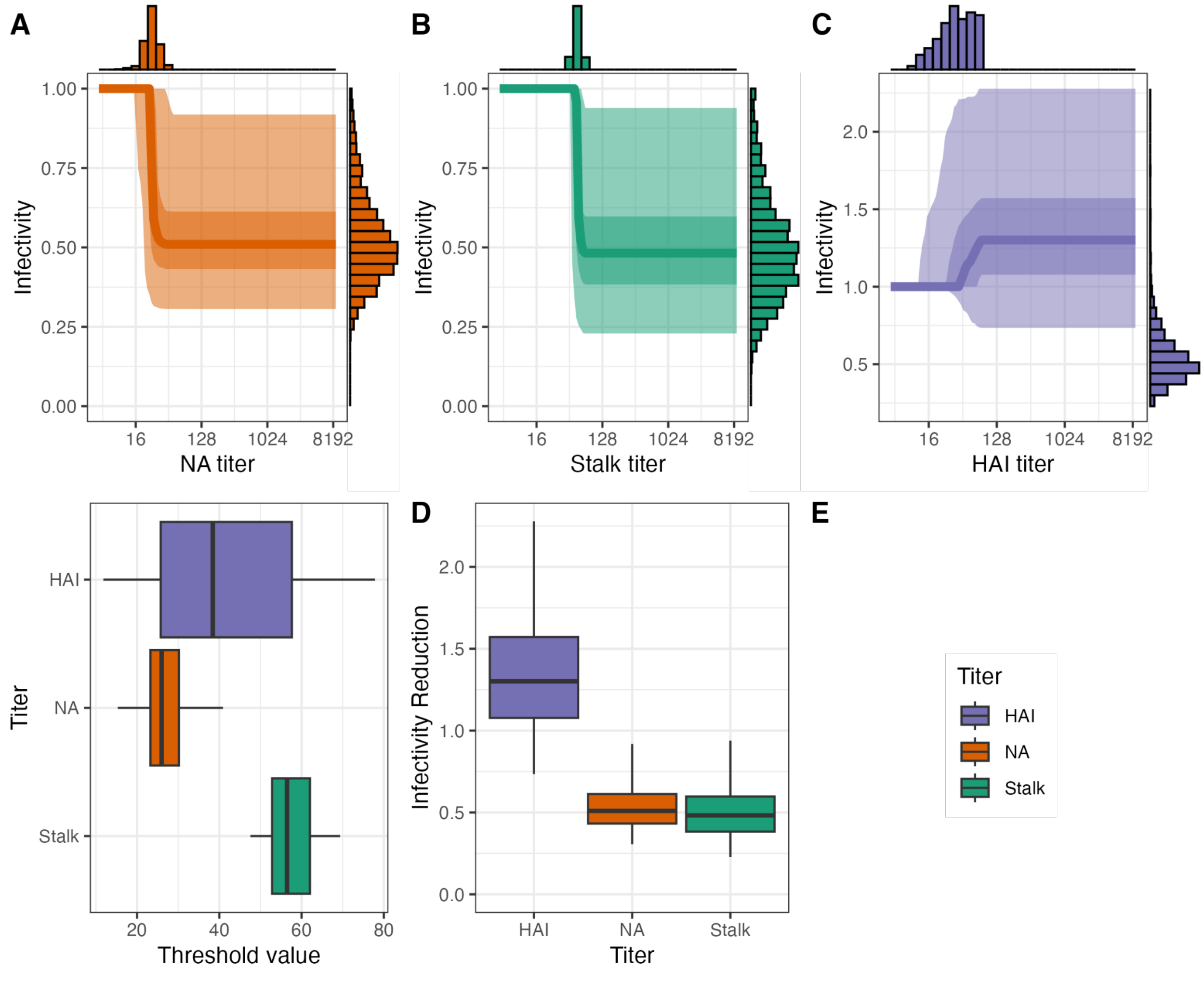

Supplementary Figure 1. Impact of pre-existing antibody titers and age on infectivity. A-C) Dose response curves to assess the infectivity relative to no detectable titers for NA, Stalk, HAI titers respectively. D) Posterior estimates of the threshold for our three titers. E) Posterior distribution of the reduction of infectivity for the three titers. The boxplots

represent these five percentiles (2.5%, 25%, 50%, 75%, and 97.5%) of the posterior distributions.

2.2 Estimates of the contact rate per household size and the relative ability of transmission of influenza during the first wave compared to the second and the third wave

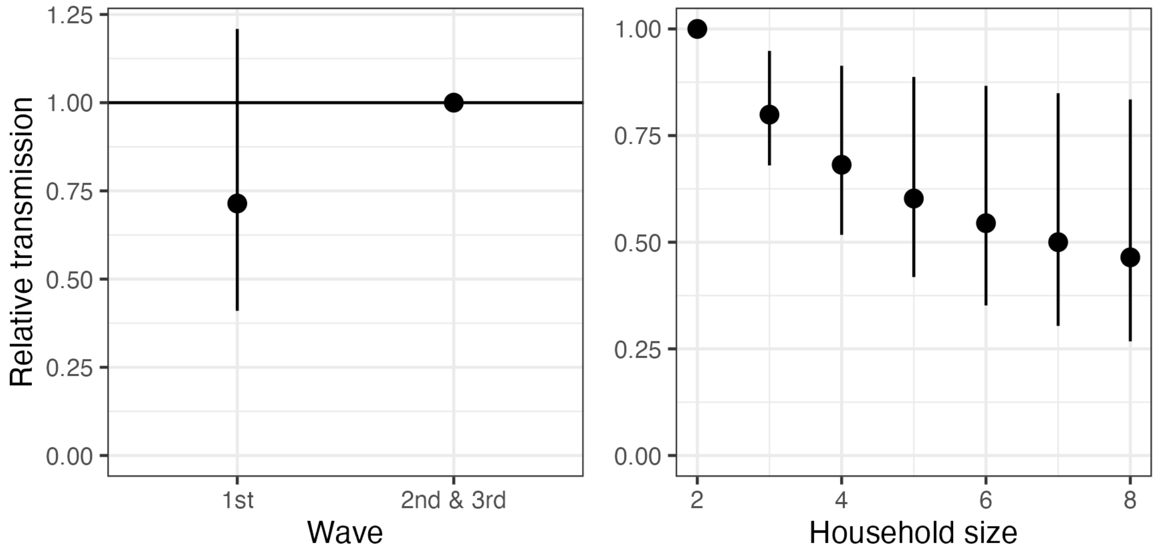

Supplementary Figure 2. Temporal and demographic variation in transmission risk A) Posterior distribution of the relative risk of transmission of the strain circulating during the first wave and the strain circulating during the subsequent waves. The dot corresponds to the median and the error bar corresponds to the 95% Credible interval. B) Estimated relative risk of transmission according to household size, relative to a household of size 2.

2.3 Testing the additive nature of the effects of different antibodies

In our baseline model, we assumed that the effects of different types of antibodies on susceptibility and infectivity were additive. In a sensitivity analysis, we assess this assumption by evaluating the combined effects of high antibody levels across multiple antigens. Using the thresholds estimated from the baseline model, we categorized individuals as having high or low antibody levels for infectivity and susceptibility analyses. For susceptibility, we evaluated the effect of: i) a single high antibody level; joint high levels of two antibodies (HA head and HA stalk; NA and HA stalk; NA and HA head); combined high levels of all three antibodies. For infectivity, we excluded HA head due to its lack of association with reduced infectivity and assessed the reduction associated with high levels of NA only, HA stalk only, or their joint high levels.

If the effects of the different types of antibodies are independent, we expect that the combined effect of the two antibodies should be equal to the product of the individual effects. This is exactly what we find here, with the estimated effect of high levels of both NA and H1 0.39 (95% CrI: 0.23–0.66) is roughly equal to the product of the effect of high NA levels (0.72; 95% CrI: 0.48–1.06) and high HA Stalk (0.53; 95% CrI: 0.21–1.11) (0.72x0.53=0.38) (Supplementary Figure S3B). The analysis of point estimates confirms the assumption of additive effects in the baseline model. The small number of individuals in some of the groups (leading sometimes to large credible intervals also indicates that our baseline model with additive effects is more appropriate.

For susceptibility (Supplementary Figure 3A), the relative susceptibility for high levels of 2 antibody titers (NA+HA Stalk: 0.27, 95% CrI: 0.16–0.45 or NA+HA Head: 0.24, 95% CrI: 0.15–0.38) is also about twice the effect of high antibody levels for a single antigen (0.52, 95% CrI: 0.34–0.75). We note large credible intervals for the effect of HA Stalk+HA Head highlighting some potential power limitations with this approach that estimates the effect of each combination of antibodies from available data. For this reason, we focus on the model assuming additive effects in our main analysis.

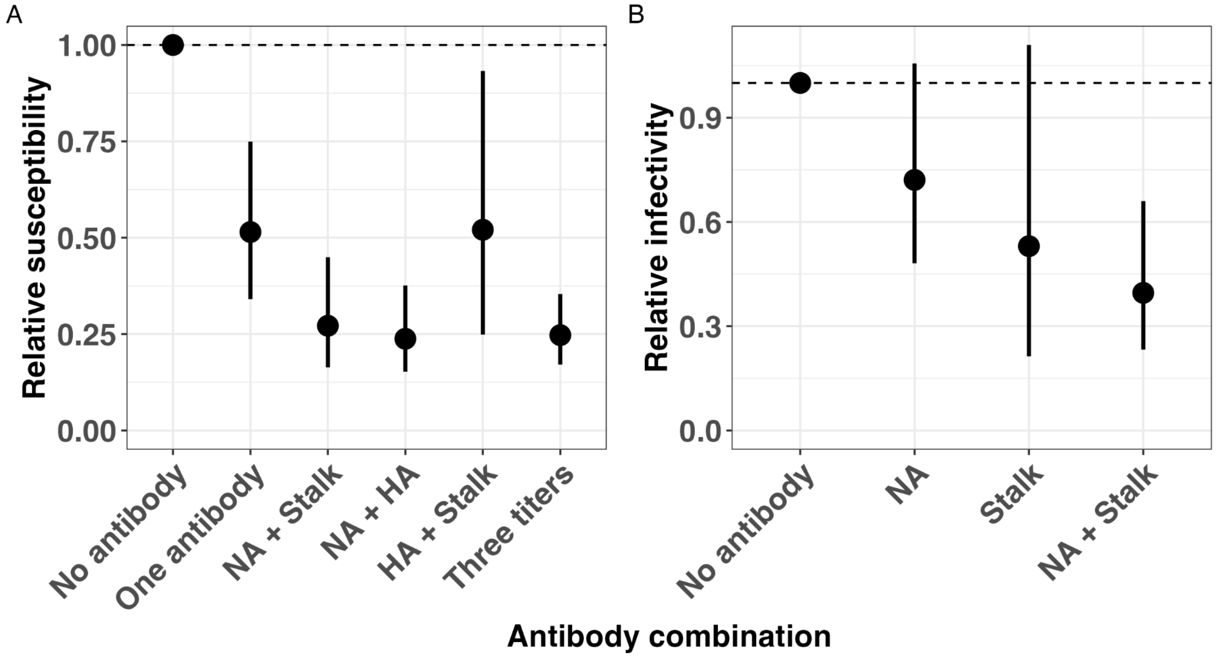

Supplementary Figure 3. Effect of Cumulative Antibody Levels on Susceptibility and Infectivity. (A) Point estimates and 95% credible intervals for relative susceptibility to A/H3N2 as a function of categorical higher-versus-lower antibody levels. Categories include zero (reference), one, each pair of antibodies, and all three antibodies combined.(B) Point estimates and 95% credible intervals for relative infectivity of A/H3N2 as a function of categorical higher-versus-lower antibody levels. Categories include zero (reference), NA, Stalk, and both antibodies combined

2.4 Assessing model adequacy comparing observed SAR and SAR within model simulations

Secondary attack rates in actual and simulated datasets, by household size. Red dots represent the observed secondary attack rates (SARs), while grey dots show SARs from simulated datasets. Violin plots illustrate the distribution of simulated SARs, with lines indicating the 2.5%, 50%, and 97.5% quantiles.

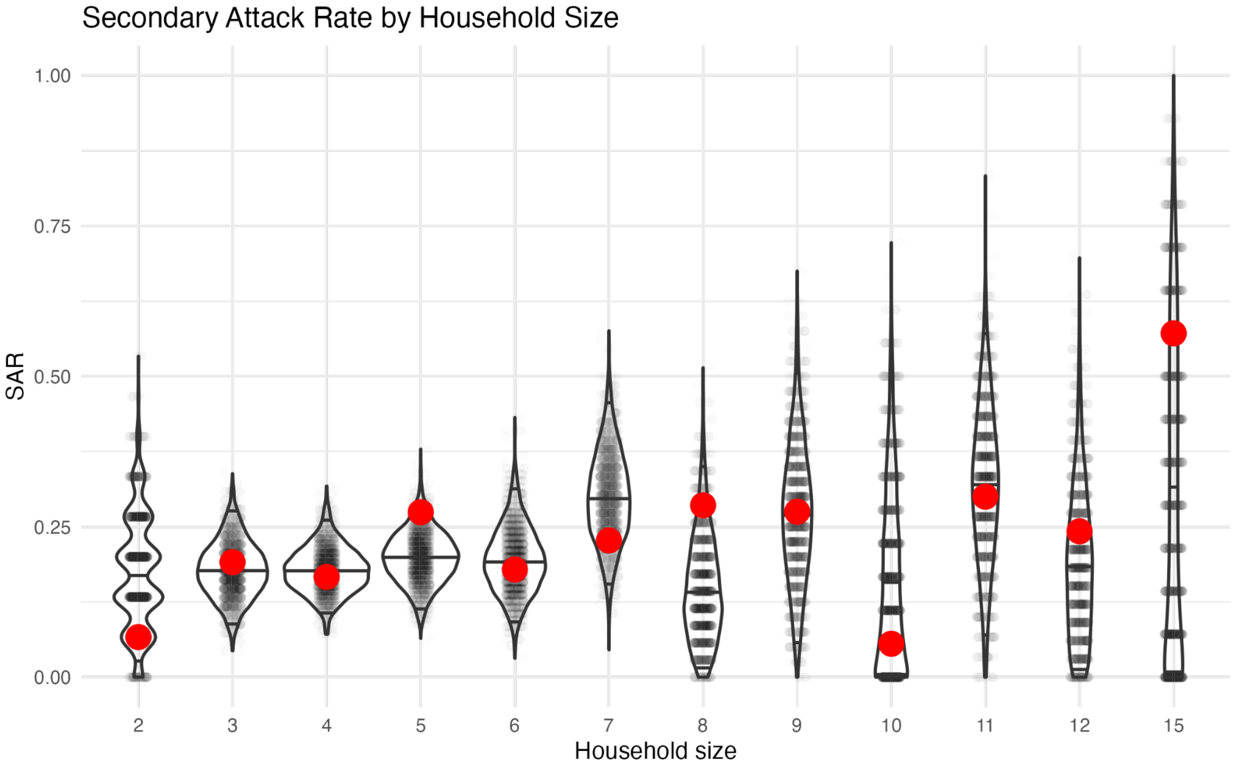

Supplementary Figure 4. Secondary attack rate in actual and simulated datasets. Secondary attack rates in actual and simulated datasets, by household size. Red dots represent the observed secondary attack rates (SARs), while grey dots show SARs from simulated datasets. Violin plots illustrate the distribution of simulated SARs, with lines indicating the 2.5%, 50%, and 97.5% quantiles.

2.5 Assessing bias in parameter estimates comparing the posterior distributions from chains on simulated datasets to the original parameter values

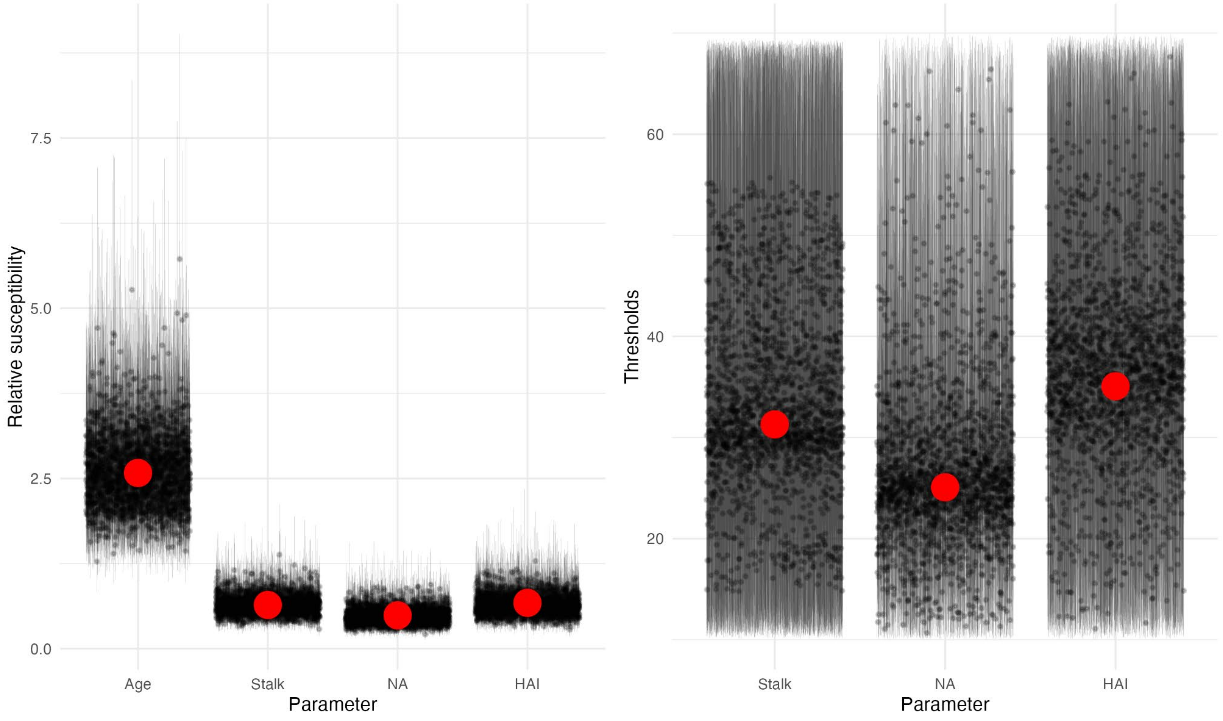

Supplementary Figure 5. Comparison of posterior susceptibility parameter distributions to original values

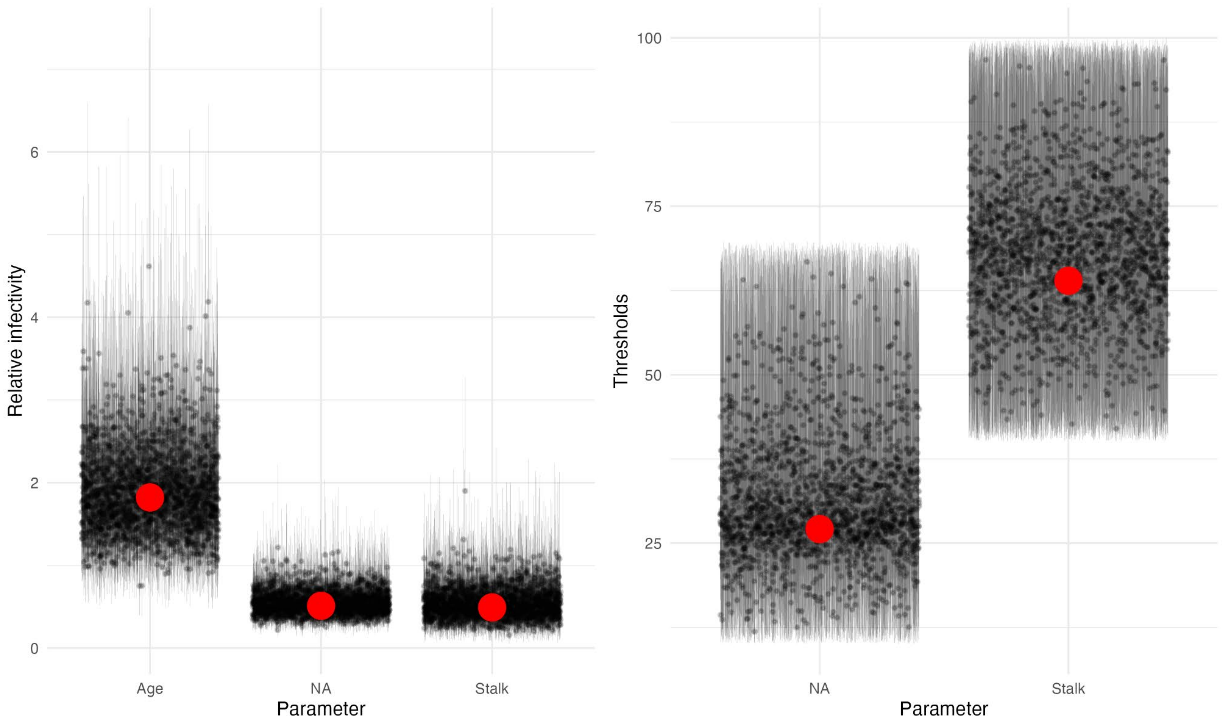

Supplementary Figure 6. Comparison of posterior infectivity parameter distributions to original values

2.6 Correlation between antibody titers through scatter plot representation

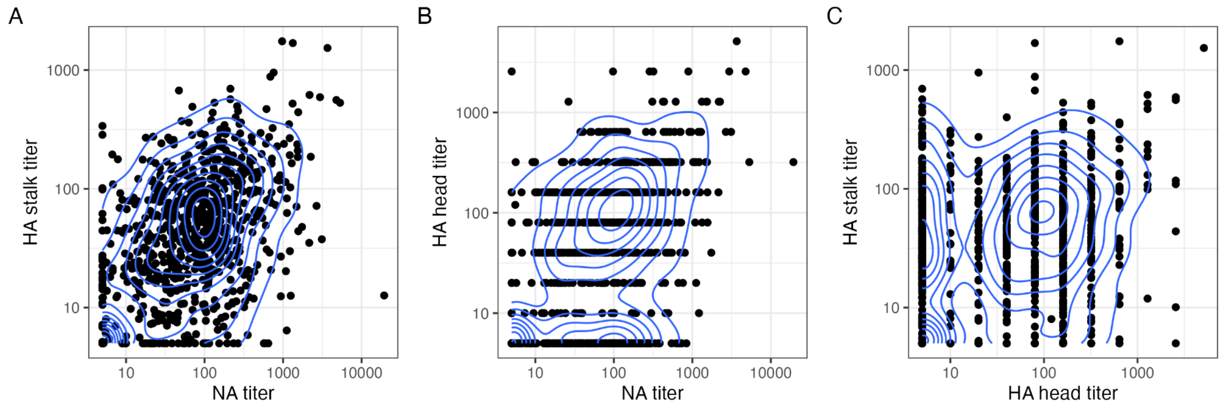

Supplementary Figure 7. Scatter plot with overlayed contour plot representing the point density of paired individual antibody titers for anti-NA antibodies by anti-HA stalk antibodies (A), anti-NA antibodies by anti-HA head antibodies (B), and anti-HA head antibodies by anti-HA stalk antibodies (C).

3 Supplementary Methods

3.1 Data considered in statistical models

Consider individual i in a household. We considered individual-level variables, including their age age_i , their pre-existing anti-HA-head antibody level $T_{HA-head_i}$, their anti-HA-stalk antibody level $T_{HA-stalk_i}$, their anti-NA antibody level T_{NA_i}, their infection status C_i (1 if infected, 0 otherwise), their symptom onset date (or test date for asymptomatic cases) O_i, their infection date X_i and the end of the follow-up period t_f. The vector of individual characteristics is denoted as $X_i = \{T^{\mathrm{i}}_{\text{HA-head}}, T^{\text{index}}_{\text{HA-stalk}}, T^{\mathrm{i}}_{\mathrm{NA}}, age^{\mathrm{i}}\}$

3.2 XGBoost Framework

3.2.1 Details about the statistical tool

To predict the probability of infection based on antibody levels and individual characteristics, we employed the XGBoost (Extreme Gradient Boosting) algorithm, a robust machine learning method well-suited for handling non-linear relationships and interactions between variables [1–3]. XGBoost operates by iteratively building an ensemble of decision trees, where each subsequent tree corrects for the residual errors of the previous ones through a gradient boosting approach. This method efficiently partitions the feature space into subregions and assigns each subspace a probability of infection.

In our model, the input features included the age and antibody titers (anti-HA head, anti-HA stalk, and anti-NA) of both the index case and the household contact.

3.2.2 The definition of the model

Considering household contact i and index case j. The XGBoost model estimates the probability of infection of i.

$$\hat{P}(C_i = 1 \mid X_i, X_j) = \sigma\left(\sum_{m=1}^{M} f_m(X_i, X_j; \theta_m)\right) \quad (1)$$

Where:

- $\hat{P}(C_i = 1 \mid X_i, X_j)$ is the predicted probability of infection given the characteristics of the index case and the contact X_i and X_j.
- $\sigma(z)$ is the logistic sigmoid function, defined as: $\sigma(z) = \dfrac{1}{1 + e^{-z}}$

This ensures that the output is a probability between 0 and 1.

- M is the total number of trees in the boosted ensemble.
- $f_m(X_i, X_j; \theta_m)$ is the prediction from the m-th decision tree, parameterized by θ_m, which includes the tree structure and leaf weights.
- The sum $\sum_{m=1}^{M} f_m(X_i, X_j; \theta_m)$ represents the aggregated predictions of all M trees in the ensemble.

XGBoost's ability to model nonlinear effects and feature interactions was particularly advantageous for capturing the complex relationships between antibody titers and the probability of infection. To prevent overfitting, we implemented a 5-fold cross-validation procedure, which splits the data into five subsets and trains the model iteratively while

ensuring validation on unseen subsets. The optimal number of boosting rounds (iterations) was determined using early stopping, which halts training once the performance on the validation set stops improving[4].

We evaluated feature importance using the mean absolute SHAP values (SHapley Additive exPlanations) ϕ_k (where k is one feature), which quantify each feature's average contribution to the prediction, independent of the direction (positive or negative).

$$\text{Mean Absolute SHAP}_j = \frac{1}{n}\sum_{i=1}^{n} |\phi_{ij}|,\ j\ one\ feature\ and\ i\ one\ observation\ (2)$$

SHAP values are derived from cooperative game theory and measure the marginal contribution of a feature to the model's predictions across all possible combinations of input features [5]. Using SHAP values, the predicted probability of infection can be expressed as :

$$\hat{P}(C_i = 1 \mid X_i, X_j) = \sigma\left(\phi_0 + \sum_{k=1}^{n} \phi_k\right)\ \ (3)$$

Where :

- ϕ_0 is the baseline prediction (average prediction when no features are included).
- ϕ_k is the SHAP value for feature X_k (of index case i or contact individual j), quantifying the marginal contribution of feature k to the prediction.
- n is the total number of features.

The statistical significance of each feature was assessed using a permutation test [6] : the observed mean absolute SHAP value was compared to a distribution generated by randomly permuting the feature values across samples. The p-value was calculated as the proportion of permutations where the permuted SHAP value exceeded the observed value, with features consistently exceeding random contributions deemed statistically significant.

$$p - value = \frac{N\{permutation\ SHAP\ >\ observed\ SHAP\}}{N\{permutation\}} \quad (4)$$

The final model offered an interpretable framework to evaluate the relationships between antibody titers, age, and infection probability. By integrating SHAP-based feature importance and statistical significance testing, we could identify the most influential factors in the model, such as specific antibody levels, and explore their relative contributions to susceptibility. This approach allows for a comprehensive understanding of how immune responses modulate infection risk.

3.3 The transmission model

3.3.1 The basal transmission model

With one infected individual i and one susceptible contact j in a household of size *n*, the instantaneous risk that case i infects susceptible contact j at time t is:

$$\lambda_{i \to j}(t|\psi) = \beta \cdot (\tfrac{5}{n})^{\delta} \cdot \kappa \cdot \rho_{\mathrm{age}_j} \cdot \mu_{\mathrm{age}_i} \cdot S(\mathrm{T}_{\mathrm{HA-head\,j}}) \cdot S(\mathrm{T}_{\mathrm{HA-stalk\,j}}) \cdot S(\mathrm{T}_{\mathrm{NA\,j}}) \cdot I(\mathrm{T}_{\mathrm{HA-head}_i}) \cdot I(\mathrm{T}_{\mathrm{HA-stalk}_i}) \cdot I(\mathrm{T}_{\mathrm{NA}_i}) \cdot g(t - t_{inf}) \quad (5)$$

ψ is the vector of the transmission parameters listed below :

- β is the scaling parameter of the household risk of infection
- κ the relative transmission rate of variants (1 during pre-Omicron period and estimated value during the Omicron period)
- $\frac{5}{n}^{\delta}$ modulates the risk of transmission based on household size. We took 5 because it was the median household size of the dataset.
- $S(T_{antibody})$ is the function linking the susceptibility to the titer $T_{antibody}$ explicit below
- $I(T_{antibody})$ is the function linking the Infectivity to the titer $T_{antibody}$
- $\rho_{\mathrm{age_j}}$ is the relative susceptibility of children (≤15 years old) compared to adults.
- $\mu_{\mathrm{age_i}}$ is the relative infectivity of children (≤15 years old) compared to adults.
- $g(t - t_{inf})$ is the generation time density defined as the interval between infector infection time t_{inf} to secondary transmission time. It is a proxy of the temporal variation of the risk of transmission following infection. It was modelled with a gamma distribution with a median of 3.5 days and sd of 2 days [7–9]

The association between the titer $T_{antibody}$ and the individual susceptibility

The susceptibility of individual i having the titer $T_{antibody_i}$ relative to no detectable antibody is :

$$S(T_{antibody_i}) = \begin{cases} 1 & \text{if } T_{antibody_i} < Ks_{antibody} \\ Rs_{antibody} & \text{if } T_{antibody_i} \geq Ks_{antibody} \end{cases} \quad (6)$$

$Rs_{antibody}$ is the susceptibility relative to titer below the threshold $Ks_{antibody}$. One threshold value $Ks_{antibody}$ and one susceptibility reduction parameter $Rs_{antibody}$ is estimated for each of the three antibodies.

The association between the titer $T_{antibody}$ and the individual infectivity

The infectivity of individual i having the titer $T_{antibody_i}$ relative to no detectable antibody is :

$$I(T_{antibody_i}) = \begin{cases} 1 & \text{if } T_{antibody_i} < Ki_{antibody} \\ Ri_{antibody} & \text{if } T_{antibody_i} \geq Ki_{antibody} \end{cases} \qquad (7)$$

$Ri_{antibody}$ is the infectivity relative to titer below the threshold $Ki_{antibody}$. One threshold value $Ki_{antibody}$ and one infectivity reduction parameter $Ri_{antibody}$ is estimated for the three antibodies.

3.3.2 Risk of infection of contact j

The total risk of infection for household contact *j* in household *k* at time *t* is dependent on the overall force of infection from all infected cases Inf_k in the household, along with the risk of community transmission α.

$$\lambda_j(t|\psi) = \alpha + \sum_{i:I_i<t} \lambda_{i\to j}(t|\psi)$$

3.3.3 The probability of transmission from the community

The probability of transmission from the community for a time period ΔT was estimated with the following formula:

$$P_{com}(\Delta T) = 1 - exp(-(\Delta T \cdot \alpha)) \quad (8)$$

3.3.4 The likelihood of the transmission process in household k

We denote Sus_k the group of not infected household contact $C_i = 0$, Inf_k the group of infected individuals and $C_i = 1$ and *i=1* is the index case of the household.

Conditional on the first infection time x_1, if we were to observe all symptom onset times $O = O_i$, infection events $X = X_i$ and infection status $C = C_i$, the likelihood of this complete transmission process in the household given transmission parameters ψ would be :

$$P(O, X, C \mid \psi) = \prod_{i \in \mathrm{Sus}_k} P(C_i = 0 \mid \psi) \prod_{i \in \mathrm{Inf}_k \setminus 1} P(X_i, C_i = 1 \mid \psi) \prod_{i \in \mathrm{Inf}_k} P(X_i \mid O_i) \; (9)$$

3.3.4.1 The contribution to the likelihood of a uninfected individuals

This is the probability for individual j to avoid infection during the whole follow up period.

$$P(C_i = 0 \mid \psi) = \exp\left(-\int_{t_0}^{t_f} \lambda_i(t)\, dt\right) \; (10)$$

3.3.4.2 The contribution to the likelihood of an infected household contact

This is the probability of escaping infection up to X_i followed by infection at X_i

$$P(X_i, C_i = 1 \mid \psi) = \left[\exp\left(-\int_{t_0}^{X_i} \lambda_i(s)\, ds\right)\right] \cdot \lambda_i(X_i) \; (11)$$

3.3.4.3 The likelihood of symptom onset and test time given infection time

- For symptomatic infected cases, the likelihood of symptom onset time O_i given infection time X_i is :

$$P(X_i|O_i) = g(O_i - X_i)$$

g(.) is the incubation period distribution modeled as a log-normal distribution of log-mean at 1 and log-sd at 1.2[10–12].

- For asymptomatic infected cases, we assume infection time to be at any time within 5 days after infection with the same probability. The likelihood of test time O_i given infection time X_i is :

$$P(X_i|O_i) = \frac{1}{5} \; if \; O_i \in [X_i; X_i + 5]$$

The transmission process and its likelihood depends on unobserved infection dates. We augmented these unobserved events from observed symptom dates by taking incubation time values ($O_i - X_i$) in the incubation period distribution (log-normal distribution of log-mean at 1 and log-sd at 1.2) for symptomatic cases and the test delay ($O_i - X_i$) within a uniform distribution ranging from 0 to 5 for asymptomatic individuals.

3.3.5 MCMC inference algorithm

We utilized a Bayesian framework with MCMC augmentation to explore the posterior distributions of transmission parameters and augmented infection times. Transmission parameters were updated using a Markov chain Monte Carlo algorithm with the Metropolis-Hastings algorithm, with log-normal proposals for positive parameters and

normal proposals for others. Proposals were tuned to ensure that the acceptance rate was between 20% and 40% for all parameters. Infection dates were augmented for each iteration of the MCMC chain.

3.3.6 Prior of the reference transmission model.

- The prior distribution for the relative susceptibility and infectivity effects of age and antibody-related factors was log-normal, with a log-mean of 0 and a log-standard deviation of 1.
- The prior risk of infection from the community α was a uniform(0,1)
- The household scaling parameter of the household risk of infection β was a uniform(0,5)
- The household size dependency parameter δ prior distribution was a Uniform(-3,3)
- The default uniform priors for antibody activation thresholds—used for both susceptibility and infectivity—were visually informed by the boosted regression tree analysis of contact infection probability (see Figure 3 of the main manuscript):
  - The default prior for susceptibility thresholds for the three antibodies was uniform(10, 70).
  - The default prior for the NA infectivity threshold was uniform(10, 40).
  - The default prior for the HA stalk infectivity threshold was uniform(40, 80).

The sensitivity of the results to these prior assumptions was assessed using alternative prior distributions, as described below.

3.3.7 Sensitivity on virus life history hypothesis and prior distributions

We conducted a series of sensitivity analyses to assess the robustness of our model estimates under varying assumptions regarding disease life history traits, including incubation period, generation time distribution, and prior assumptions on susceptibility and infectivity possible value and possible values of antibody thresholds at which the effect is significant.

3.3.7.1 Disease Life History Traits

We tested the following condition concerning disease life history traits :

- Incubation period was varied from lognormal with mean=1 and sd = 1.2 to mean= 0.6 days and SD = 0.8 and mean=2 days SD = 1.6.
- Generation time varied from a gamma of mean 3.5 and sd 2 to a mean of 3.0 days and sd = 1.5 and a mean of 4.0 days and sd = 2.0.
- The default model estimates were compared against these variations, and parameter estimates were evaluated for significant deviations.

3.3.7.2 Sensitivity Analysis on Prior Assumptions

To assess the robustness of prior distributions, we tested different assumptions regarding susceptibility ratios and antibody threshold definitions:

- Prior distributions for susceptibility and infectivity factors were evaluated using SD = 2.0.

- We tested uniform priors centered around the target thresholds with varying widths of 20, 40, 60, and 80. For example, with a width of 20, the priors were set to 25–45 for susceptibility, 20–40 for NA infectivity, and 50–70 for HA stalk infectivity.

Each sensitivity test involved re-estimating key parameters and comparing them to the default model estimates. The results remained largely stable across conditions, with minor variations in parameter estimates for NA infectivity and Stalk susceptibility, suggesting that these factors are more sensitive to changes in disease life history traits and prior assumptions.

All sensitivity analysis results are summarized in Supplementary Tables 2 and 3.

3.3.8 Programming

The MCMC algorithm was coded in C++ using Visual Studio Code version 1.89.1. Chains were run for 50,000 iterations and recorded every 20 iterations. Posterior parameter distributions were sampled from the MCMC after discarding a burn-in of 10000 iterations. Acceptance rates and convergence was assessed visually. The XGboost regression analysis was done with Python 3.13.0

References

1. Viejo, G., Cortier, T. & Peyrache, A. Brain-state invariant thalamo-cortical coordination

revealed by non-linear encoders. *PLoS Comput. Biol.* **14**, e1006041 (2018).

2. Freund, Y. & Schapire, R. E. A decision-theoretic generalization of on-line learning and an application to boosting. *J. Comput. Syst. Sci.* **55**, 119–139 (1997).
3. Schapire, R. E. The boosting approach to machine learning: An overview. in *Nonlinear Estimation and Classification* 149–171 (Springer New York, New York, NY, 2003).
4. Vabalas, A., Gowen, E., Poliakoff, E. & Casson, A. J. Machine learning algorithm validation with a limited sample size. *PLoS One* **14**, e0224365 (2019).
5. Lundberg, S. & Lee, S.-I. A unified approach to interpreting model predictions. *arXiv [cs.AI]* (2017).
6. Niknian, M. Permutation tests: A practical guide to resampling methods for testing hypotheses. *Technometrics* **37**, 341–342 (1995).
7. Cauchemez, S., Carrat, F., Viboud, C., Valleron, A. J. & Boëlle, P. Y. A Bayesian MCMC approach to study transmission of influenza: application to household longitudinal data. *Stat. Med.* **23**, 3469–3487 (2004).
8. Ghani, A. *et al.* The early transmission dynamics of H1N1pdm influenza in the United Kingdom. *PLoS Curr.* **1**, RRN1130 (2009).
9. Carrat, F. *et al.* Time lines of infection and disease in human influenza: a review of volunteer challenge studies. *Am. J. Epidemiol.* **167**, 775–785 (2008).
10. Cauchemez, S. *et al.* Household transmission of 2009 pandemic influenza A (H1N1) virus in the United States. *N. Engl. J. Med.* **361**, 2619–2627 (2009).
11. Nishiura, H. & Inaba, H. Estimation of the incubation period of influenza A (H1N1-2009) among imported cases: addressing censoring using outbreak data at the origin of importation. *J. Theor. Biol.* **272**, 123–130 (2011).
12. Nishiura, H. Early efforts in modeling the incubation period of infectious diseases with an acute course of illness. *Emerg. Themes Epidemiol.* **4**, 2 (2007).
