# Supplementary material for: Anti-neuraminidase and anti-HA stalk antibodies reduce the susceptibility to and infectivity of influenza A/H3N2 virus
Source: Nat Commun. 2025 Dec 11;16:10910. doi: 10.1038/s41467-025-65283-0 (PMC12698684; doi:10.1038/s41467-025-65283-0)
Supplement: Supplementary file 3 — Reporting Summary [file 41467_2025_65283_MOESM3_ESM.pdf]

nature portfolio

Corresponding author(s): Gordon, Aubree

Last updated by author(s): Sep 22, 2025

Reporting Summary

Nature Portfolio wishes to improve the reproducibility of the work that we publish. This form provides structure for consistency and transparency in reporting. For further information on Nature Portfolio policies, see our Editorial Policies and the Editorial Policy Checklist.

Statistics

For all statistical analyses, confirm that the following items are present in the figure legend, table legend, main text, or Methods section.

n/a	Confirmed	
☐	☒	The exact sample size (*n*) for each experimental group/condition, given as a discrete number and unit of measurement
☐	☒	A statement on whether measurements were taken from distinct samples or whether the same sample was measured repeatedly
☐	☒	The statistical test(s) used AND whether they are one- or two-sided<br>*Only common tests should be described solely by name; describe more complex techniques in the Methods section.*
☐	☒	A description of all covariates tested
☐	☒	A description of any assumptions or corrections, such as tests of normality and adjustment for multiple comparisons
☐	☒	A full description of the statistical parameters including central tendency (e.g. means) or other basic estimates (e.g. regression coefficient) AND variation (e.g. standard deviation) or associated estimates of uncertainty (e.g. confidence intervals)
☐	☒	For null hypothesis testing, the test statistic (e.g. *F*, *t*, *r*) with confidence intervals, effect sizes, degrees of freedom and *P* value noted<br>*Give P values as exact values whenever suitable.*
☐	☒	For Bayesian analysis, information on the choice of priors and Markov chain Monte Carlo settings
☒	☐	For hierarchical and complex designs, identification of the appropriate level for tests and full reporting of outcomes
☒	☐	Estimates of effect sizes (e.g. Cohen's *d*, Pearson's *r*), indicating how they were calculated

Our web collection on statistics for biologists contains articles on many of the points above.

Software and code

Policy information about availability of computer code

Data collection	No software was used in the collection of data.
Data analysis	Analyses were conducted using R version 4.2.1-4.5.0 and Visual Studio Code version 1.82.0-1.104

For manuscripts utilizing custom algorithms or software that are central to the research but not yet described in published literature, software must be made available to editors and reviewers. We strongly encourage code deposition in a community repository (e.g. GitHub). See the Nature Portfolio guidelines for submitting code & software for further information.

Data

Policy information about availability of data

All manuscripts must include a data availability statement. This statement should provide the following information, where applicable:

- Accession codes, unique identifiers, or web links for publicly available datasets
- A description of any restrictions on data availability
- For clinical datasets or third party data, please ensure that the statement adheres to our policy

Researchers interested in accessing the study data are encouraged to submit a formal request to A.G. or the Health Sciences and Behavioral Sciences Institutional Review Board at the University of Michigan. To uphold ethical standards and ensure appropriate data use, each request will undergo a case-by-case review and approval process. Additionally, as the data include information collected in Nicaragua, access is subject to Nicaraguan data ownership regulations and may require approval from relevant Nicaraguan authorities. Final approval is expected within two months, and if access is granted, duration of data access will be granted on a case-by-case basis.

Research involving human participants, their data, or biological material

Policy information about studies with human participants or human data. See also policy information about sex, gender (identity/presentation), and sexual orientation and race, ethnicity and racism.

Reporting on sex and gender	Both biologic males and biologic females are included in this research. The work was not stratified by biologic sex or gender, as the authors believe that the findings are applicable to both groups with a low likelihood of effect modification by gender/sex.
Reporting on race, ethnicity, or other socially relevant groupings	Neither race nor ethnicity are discussed in this manuscript; this study is based in Managua, Nicaragua, and the population is homogeneous with respect to ethnicity and race. Descriptions of the study population are available in the manuscript.
Population characteristics	The population consists of all age groups; 4.3% of the population is 0-1 year of age, 11.7% of the population is 2-4 years of age, 32% of the population is 5-14 years of age, and 52% of the population is 15 years of age or older, with an average age of 22.3 years. The population is largely unvaccinated for influenza, with 0.2% of the population having been vaccinated in the 6 months prior to their respective intensive monitoring period. The population is drawn from District II of Managua, Nicaragua, which predominantly consists of low-to-middle-class neighborhoods with access to electricity and city water.
Recruitment	This study uses data from two household influenza transmission studies based in Managua, Nicaragua: the Household Influenza Transmission Study (HITS) and the Household Influenza Cohort Study (HICS). HITS is a case-ascertained study, meaning that influenza-positive individuals are identified, and other members of their household recruited for enrollment, that ran from 2012 to 2017, and HICS is a prospective household-based cohort study that began in 2017 and is currently ongoing. In both studies, influenza A/H3N2 virus-positive individuals, the index cases, are initially detected at a health center, where household members are enrolled (HITS) or activated (HICS) into intensive monitoring for a period of ~14 days.
Ethics oversight	These studies were approved by the institutional review boards at the Nicaraguan Ministry of Health and the University of Michigan and are in accordance with the Helsinki Declaration of the World Medical Association. Written consent to participate or parental permission was obtained for all participants; in children older than 6 years, verbal assent was obtained.

Note that full information on the approval of the study protocol must also be provided in the manuscript.

Field-specific reporting

Please select the one below that is the best fit for your research. If you are not sure, read the appropriate sections before making your selection.

☐ Life sciences ☒ Behavioural & social sciences ☐ Ecological, evolutionary & environmental sciences

For a reference copy of the document with all sections, see nature.com/documents/nr-reporting-summary-flat.pdf

Behavioural & social sciences study design

All studies must disclose on these points even when the disclosure is negative.

Study description	This is a quantitative study utilizing data from a case-ascertained study (HITS) and a prospective household-based cohort study with an embedded transmission study.
Research sample	The research sample consists of individuals from District II in Managua, NIcaragua, which which predominantly consists of low-to-middle-class neighborhoods with access to electricity and city water.
Sampling strategy	Individuals enrolled in the HITS were sampled according to visits to the Health Center Sócrates Flores Vivas; all individuals at HCSFV were eligible for enrollment so long as they a) they had influenza-like illness, defined as fever or feverishness with cough, sore throat, or runny nose; 2) their symptom onset, defined as the earliest day with influenza-like illness, was within the previous 48 hours; 3) they were positive for influenza by rapid antigen test or reverse transcription PCR (RT-PCR); 4) no household members had had symptoms of influenza-like illness in the previous 2 weeks; and 5) they lived with >1 additional person. HICS households were enrolled from individuals who had participated in the HITS. Sample size calculations were not performed, as all individuals in households activated for influenza A/H3N2 virus infection in the three most recent A/H3N2-dominated seasons were included (sans exclusions, see below).
Data collection	Oral/pharyngeal swabs were collected through home visits by dedicated study personnel, stored at 4°C–8°C and transported them to the National Virology Laboratory (Managua, Nicaragua) within 12 hours. We tested all samples for influenza on an ABI 7500 Fast PCR platform (Applied Biosystems, Foster City, CA, USA) following validated protocols from the US Centers for Disease Control and Prevention. Survey data, such as demographics and symptoms, were collected by study personnel either using pen and paper or through an app-based interface.
Timing	Samples in this work include three influenza seasons: 2014 (samples included were collected between September 1st 2014 and December 31st 2014), 2016 (samples included were collected between October 15th, 2016 and January 15, 2017), and 2017 (samples included were collected between July 2017 and January 15, 2017). Sample collection timing depended on influenza positivity timing for each of the seasons.
Data exclusions	899 participants were originally assessed for inclusion; of these, 835 were ultimately included, with 64 individuals excluded.

	Exclusions were made only for individuals who had data missing that were necessary for the proper function of the transmission model, such as onset date or positive test date.
Non-participation	All participants in this study population fully participated in the study without attrition.
Randomization	Participants were not randomly allocated into experimental groups in this study.

Reporting for specific materials, systems and methods

We require information from authors about some types of materials, experimental systems and methods used in many studies. Here, indicate whether each material, system or method listed is relevant to your study. If you are not sure if a list item applies to your research, read the appropriate section before selecting a response.

Materials & experimental systems

n/a	Involved in the study
☐	☒ Antibodies
☒	☐ Eukaryotic cell lines
☒	☐ Palaeontology and archaeology
☒	☐ Animals and other organisms
☒	☐ Clinical data
☒	☐ Dual use research of concern
☒	☐ Plants

Methods

n/a	Involved in the study
☒	☐ ChIP-seq
☒	☐ Flow cytometry
☒	☐ MRI-based neuroimaging

Antibodies

Antibodies used	Anti-human IgG (Fab-specific)-HRP (Sigma-Aldrich, A0293; 1:3000) diluted in 1% milk-PBS-T was added (50 µL/well) and incubated for 1 hour at RT.
Validation	After plates were washed three times with PBS-T, 100 µL/well of o-phenylenediamine dihydrochloride (OPD; SIGMAFAST) substrate was added. The reaction was stopped after 10 minutes with 50 µL/well of 3 M HCl (ThermoFisher). Optical density (OD) at 490 nm was measured using a BioTek Synergy H1 or Synergy 4 plate reader.<br>Area under the curve (AUC) was calculated to quantify total antibody binding across serial dilutions. OD values at each dilution were plotted against dilution factor, and the AUC was determined by integrating the curve using GraphPad Prism software

Plants

Seed stocks	*Report on the source of all seed stocks or other plant material used. If applicable, state the seed stock centre and catalogue number. If plant specimens were collected from the field, describe the collection location, date and sampling procedures.*
Novel plant genotypes	*Describe the methods by which all novel plant genotypes were produced. This includes those generated by transgenic approaches, gene editing, chemical/radiation-based mutagenesis and hybridization. For transgenic lines, describe the transformation method, the number of independent lines analyzed and the generation upon which experiments were performed. For gene-edited lines, describe the editor used, the endogenous sequence targeted for editing, the targeting guide RNA sequence (if applicable) and how the editor was applied.*
Authentication	*Describe any authentication procedures for each seed stock used or novel genotype generated. Describe any experiments used to assess the effect of a mutation and, where applicable, how potential secondary effects (e.g. second site T-DNA insertions, mosiacism, off-target gene editing) were examined.*
